# Epigenetic clock analyses of cellular senescence and ageing

**DOI:** 10.18632/oncotarget.7383

**Published:** 2016-02-14

**Authors:** Donna Lowe, Steve Horvath, Kenneth Raj

**Affiliations:** ^1^ Radiation Effects Department, Centre for Radiation, Chemical and Environmental Hazards, Public Health England, Chilton, Didcot, Oxfordshire, OX11 0RQ, United Kingdom; ^2^ Human Genetics and Biostatistics, David Geffen School of Medicine, University of California, Los Angeles, Los Angeles, CA, USA

**Keywords:** DNA methylation, ageing, senescence, DNA damage, radiation, Gerotarget

## Abstract

A confounding aspect of biological ageing is the nature and role of senescent cells. It is unclear whether the three major types of cellular senescence, namely replicative senescence, oncogene-induced senescence and DNA damage-induced senescence are descriptions of the same phenomenon instigated by different sources, or if each of these is distinct, and how they are associated with ageing. Recently, we devised an epigenetic clock with unprecedented accuracy and precision based on very specific DNA methylation changes that occur in function of age. Using primary cells, telomerase-expressing cells and oncogene-expressing cells of the same genetic background, we show that induction of replicative senescence (RS) and oncogene-induced senescence (OIS) are accompanied by ageing of the cell. However, senescence induced by DNA damage is not, even though RS and OIS activate the cellular DNA damage response pathway, highlighting the independence of senescence from cellular ageing. Consistent with this, we observed that telomerase-immortalised cells aged in culture without having been treated with any senescence inducers or DNA-damaging agents, re-affirming the independence of the process of ageing from telomeres and senescence. Collectively, our results reveal that cellular ageing is distinct from cellular senescence and independent of DNA damage response and telomere length.

## INTRODUCTION

While ageing at the level of the organism is obvious and easily understood, the biological aspect of ageing is far from clear. Even the definition of ageing is not self-evident. It is reasonable to consider ageing as a natural biological process that in time, leads to the eventual failure of organs, as it is this that gives rise to the phenotypic features of ageing; from the benign, such as thinning of the skin and greying of the hair, to the pathological, such as cataracts and cardiovascular disease. Understanding why tissues and cells function sub-optimally and eventually fail in time, will greatly aid our understanding of ageing.

One model of ageing posits that the failure of tissues to function properly is due to the depletion of stem cells [1]. Stem cells, which are the reservoir cells of tissues, may have finite capacities of proliferation such as being limited by the lengths of their telomeres. Their eventual depletion leads to the deficit of properly functioning cells, causing phenotypic changes that constitute ageing. While this model is plausible and supported by strong circumstantial evidence, it is presently difficult to prove or refute directly, not least because the identification of specific tissue stem cells is difficult. Similarly, the association between telomere length and ageing, although widely reported, is not without inconsistencies [2-4].

There is however, another model of ageing which is based on the observation that the number of senescent cells in the body increases in function of organism age [5-7]. While this could be interpreted to mean that senescent cells cause ageing, it could also equally mean that senescent cells are a consequence of ageing. In this regard, it is noteworthy that there is increasing evidence to demonstrate that senescent cells are not benign. Instead they secrete bio-chemicals that are detrimental to normal functioning of neighbouring cells. The senescence-associated secretory phenotype (SASP) proteins include cytokine, chemokines and proteases [8, 9] and their paracrine activities are very diverse and include oncogenic characteristics that stimulate cellular proliferation and epithelial-mesenchymal transition. Importantly, SASP proteins also promote chronic inflammation, which is the origin of almost all age-related pathologies [10, 11]. As such, SASP proteins, through their different effects on normal and cancer cells, induce deterioration of the tissue [12, 13]. Recently, Baker et al. [14] demonstrated that removal of senescent cells in mice delays ageing-associated disorders, providing very strong support for the notion that senescent cells mediate the effects of ageing. Hence it follows that to understand ageing, it is necessary to understand cellular senescence. This model of active induction of ageing (via senescent cells) does not exclude the role of stem cell depletion described above, which could indeed be a result of stem cell senescence.

At present, the causes of cellular senescence *in vivo* are not known for certain but *in vitro*, cells can become senescent through (i) telomere shortening via exhaustive replication (replicative senescence)[15], (ii) over-expression of oncogene [16-18] or (iii) DNA damage [19]. While it is easy to perceive replicative senescence (RS) as part of a *bona fide* mechanism of ageing, it is more challenging to consider oncogene-induced senescence (OIS) as a significant contributor to natural ageing. Instead OIS has been proposed to function as a tumour suppressor mechanism. The only obvious common factor between RS and OIS is the co-opting of the DNA damage signalling mechanism to usher cells into arrest. This is because when telomeres become shorter than the critical length and cannot be properly protected by telomere proteins, they are detected as damaged DNA by the cell [20]. Similarly, ectopic expression of oncogene causes aberrant re-initiation of DNA replication, the products of which are sensed by the cell as damaged DNA [21, 22]. It appears that the constant presence of either telomere ends or abnormal DNA structures of replication activates the DNA damage signalling pathway perpetually, initiating the process of cellular senescence.

Another age-associated change that occurs to the human genome is the level of cytosine methylation at some CpG sites [23-32]. These changes have recently been measured and found to be associated very precisely with age [33-35]. Recently, we developed a multivariate estimator of chronological age, referred to as epigenetic clock, based on methylation levels of 353 CpGs [34]. The following features of this clock demonstrates that its age estimates capture several aspects of biological age: (a) it can accurately measure the age of cells regardless of tissue types including brain, liver, kidney, breast and lung [34] (b) its accuracy (r = 0.96 on subjects aged between 0 to 100 and r = 0.77 in middle age subjects) is substantially higher than that of other molecular markers such as telomere length (r = 0.5) [36] (c) it is able to predict mortality independent of health, life-style or genetic factors [37] (d) its measurements correlate with cognitive and physical fitness amongst the elderly [38] and (e) it is able to detect accelerated ageing induced by various factors including obesity [39], Down syndrome [40] and HIV infection [41]. Here, we apply this epigenetic clock to study the relationship between ageing and senescence of isogenic cells induced by exhaustive replication, ectopic oncogene over-expression or radiation-induced DNA damage.

## RESULTS AND DISCUSSION

**Figure 1:**
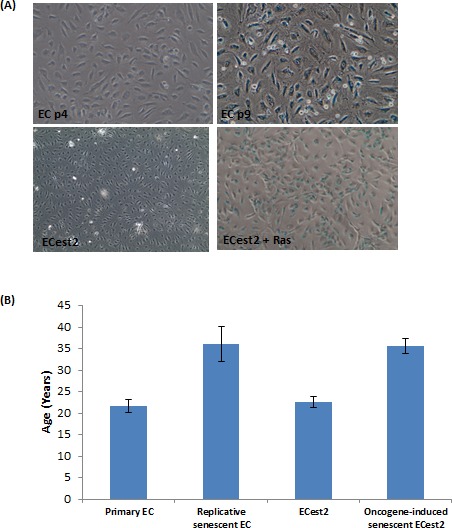
Replicative senescence and oncogene-induced senescence are accompanied by biological ageing **A.** Staining of cells for expression of senescence-associated beta galactosidase, in blue. Upper panel cells visualised with 10X objective and lower panel cells with 4X objective. The sizes of senescent cells are significantly bigger than the non-senescent cells. **B.** Epigenetic clock measurements of biological ages of cellular DNA. Each bar represents three biological replicates.

To determine whether senescent cells alter their DNA methylation state in an age-dependent manner, we used primary endothelial cells (ECs) that were derived from the human coronary artery of a 19 year old male. Some of these cells were cultured with repeated passaging for 5-6 weeks until they were no longer able to proliferate, increased in size and began expressing senescence-associated beta-galactosidase (SA-beta gal) (Figure 1A), indicating that they have undergone replicative senescence. Analyses of their DNA by the previously described epigenetic clock [34], revealed that the replicative senescent cells have indeed aged. To test whether senescence induced by oncogene over-expression also undergo ageing, a subset of ECs were first immortalised with est2, a yeast homologue of hTERT. This is to ensure that when they were subsequently transduced with the rasV12 oncogene, their resulting senescence would not be due to replicative senescence brought on by telomere attrition. As can be seen in Figure 1B, immortalisation by telomerase *per se* did not alter the epigenetic age of the cells (compare Primary EC and ECest2), but when they were transduced with the rasV12 gene, they became senescent within 10 days (Figure 1A) and their DNA exhibited epigenetic ageing. Collectively, the results in Figure 1B appear to suggest the existence of a connection between senescent cells and epigenetic ageing.

**Figure 2:**
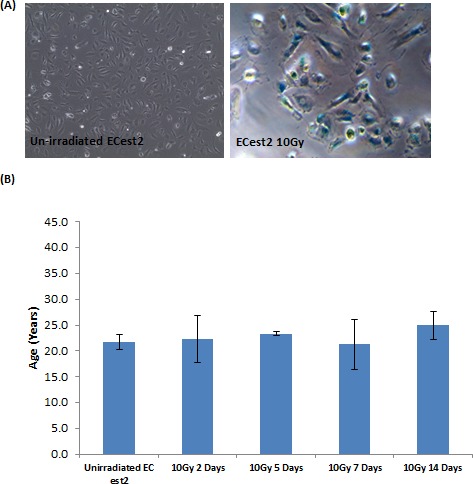
DNA damage-induced senescence does not induce biological ageing **A.** Staining of cells for expression of senescence-associated beta galactosidase, in blue. Cells visualised with 10X objective. **B.** Epigenetic clock measurements of biological ages of cellular DNA. Each bar represents three biological replicates.

While it may be intuitive that replicative senescent cells, which have undergone numerous rounds of continuous proliferation in 5-6 weeks, exhibited epigenetic ageing (albeit in an accelerated way), it is surprising that rasV12-expressing cells, which senesced within 10 days also aged. Although these two senescent states are induced very differently, they share a common cellular mechanism which they use to arrest their cycle; the DNA damage response pathway. Hence, it would stand to reason that prolonged activation of cellular DNA damage response may be the cause of senescence and epigenetic ageing. Indeed, induction of DNA damage signalling by irradiating cells with 10Gy of X-ray induced them to senesce (Figure 2A) [42]. However, when the DNA of these cells were analysed at various time-points post-irradiation, they were found not to have aged (Figure 2B). This surprising effect does not represent an experimental artefact since we could replicate this observation in two publicly available data sets [43, 44] (data not shown). Clearly, DNA damage response is not coupled to epigenetic ageing and that RS and OIS induce cellular ageing through a mechanism that is not part of the DNA damage response pathway. The lack of effect on ageing by radiation-induced senescence demonstrates that the association between senescence and epigenetic ageing is more nuanced, in that it is an independent association, as opposed to an inextricable mechanistic one.

**Figure 3:**
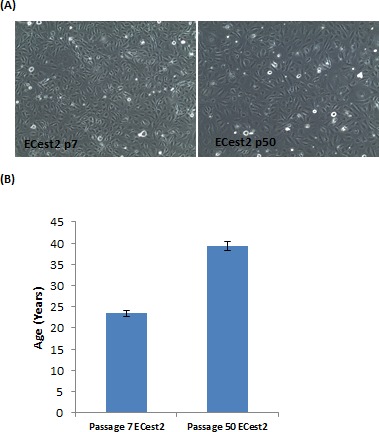
Ageing of telomerase-expressing cells in culture **A.** Staining of cells for expression of senescence-associated beta galactosidase, in blue. Cells visualised with 10X objective. **B.** Epigenetic clock measurements of biological ages of cellular DNA. Each bar represents three biological replicates.

This point is reinforced in a separate, yet conceptually connected experiment. Analyses of cells immortalised by telomerase showed late (p50) passage cells to have aged, even without having been subjected to any known senescence inducers (Figure 3). These cells continue to proliferate in culture beyond passage 50 and do not exhibit any signs of senescence, demonstrating that the process of cellular ageing continues unabated in cells whose telomeres were maintained. This shows that removal of the inducers of senescence does not halt ageing, once again underlining the fact that cellular ageing is a process that is distinct from senescence.

Collectively, these two sets of observation make an effective case for the uncoupling of senescence from cellular ageing. This however, appears at first sight to be inconsistent with the fact that senescent cells contribute to the physical manifestation of organism ageing, as demonstrated elegantly by Baker et al., where removal of senescent cells slowed down ageing. In the light of our observations however, it is proposed that cellular senescence is a state that cells are forced into as a result of external pressures such as DNA damage, ectopic oncogene expression and exhaustive proliferation of cells to replenish those eliminated by external/environmental factors. These senescent cells, in sufficient numbers, will undoubtedly cause the deterioration of tissues, which is interpreted as organism ageing. However, at the cellular level, ageing, as measured by the epigenetic clock, is distinct from senescence. It is an intrinsic mechanism that exists from the birth of the cell and continues. This implies that if cells are not shunted into senescence by the external pressures described above, they would still continue to age. This is consistent with the fact that mice with naturally long telomeres still age and eventually die even though their telomere lengths are far longer than the critical limit, and they age prematurely when their telomeres are forcibly shortened, due to replicative senescence. Hence senescence is a route by which cells exit prematurely from the natural course of cellular ageing.

Finally, it is necessary to address specifically the role of telomeres as it is easy to confound them with cellular ageing because at first view, they appear to share similar features. Since critical telomere length is attained after many rounds of proliferation, which takes a long time and hence occurs later in life, it is easy to mistake this for a functional link with age even though telomere length has only a modest correlation with chronological age (r = 0.5), while cellular ageing as measured by the epigenetic clock has a far higher degree of association (0.99 for solid tissues) with biological ageing. The fact that maintenance of telomere length by telomerase did not prevent cellular ageing defines the singular role of telomeres as that of a means by which cells restrict their proliferation to a certain number; which was the function originally ascribed to it. Cellular ageing on the other hand proceeds regardless of telomere length.

Although the characteristics of cellular ageing are still not well known, the remarkable precision with which the epigenetic clock can measure it and correlate it to biological ageing remove any doubt of its existence, distinctiveness and importance. This inevitably raises the question of what is the nature of this cellular ageing, and what are its eventual physical consequences. Admittedly, the observations above do not purport to provide the answer, but they have however, cleared the path to its discovery by unshackling cellular ageing from senescence, telomeres and DNA damage response, hence inviting fresh perspectives into its possible mechanism. In summary, the results from these experiments, while apparently simple in their presentation, untangles a conceptual knot that hitherto tied senescence, DNA damage signalling, ageing and telomeres together in an incomprehensible way. Here we propose that cellular ageing, as measured by the epigenetic clock, is an intrinsic property of cells, and while independent, its speed can be affected by some factors; a feature that would undoubtedly be exploited to characterise and elucidate its mechanism.

## MATERIALS AND METHODS

### Immortalisation of primary endothelial cells from human coronary artery

ECs from human coronary artery were purchased from European Cell Culture Collection (HCAECs Cat. No: 300-05) and transduced with retroviruses bearing the est2 gene; a yeast homologue of the human TERT protein. The resulting cells (ECest2) were cultured in HCAEC media from the same provider. All cells were mycoplasma-free and cultured at 37°C with 5% carbon dioxide. To generate rasV12 -expressing cells, ECest2 were infected with recombinant retroviruses bearing the gene (pBabePurorasV12). Control cells were infected with pBabePuro.

### DNA extraction

Cells were harvested by trypsinisation and cell pellets were subjected to treatments according to the protocol provided by the QIAamp mini DNA extraction kit (Qiagen: Cat no:51304)

### Illumina 450 analyses

DNA was analysed by NxT-DX (Belgium) using the Illumina 450 system

### Senescence-associated beta-galactosidase assay

This assay was carried out using the Senescence β-Galactosidase Staining Kit 9860 from Cell Signaling Technology according to the manufacturer's instructions.

### DNA methylation age and the epigenetic clock

The epigenetic clock is defined as a prediction method of age based on the linear combination of the DNA methylation levels of 353 CpGs dinucleotides [34]. Predicted age, referred to as DNA methylation age, correlates with chronological age in sorted cell types (CD4 T cells, monocytes, B cells, glial cells, neurons) and tissues and organs including whole blood, brain, breast, kidney, liver, lung, saliva [34]. By construction, the epigenetic clock (and software) applies to data generated using either the Illumina 450K or the 27K platform. Mathematical details and software tutorials for the epigenetic clock can be found in the Additional files of [34]. An online age calculator can be found at our webpage: http://labs.genetics.ucla.edu/horvath/dnamage/.
